# A Requirement of TolC and MDR Efflux Pumps for Acid Adaptation and GadAB Induction in *Escherichia coli*

**DOI:** 10.1371/journal.pone.0018960

**Published:** 2011-04-26

**Authors:** Kari N. W. Deininger, Akina Horikawa, Ryan D. Kitko, Ryoko Tatsumi, Judah L. Rosner, Masaaki Wachi, Joan L. Slonczewski

**Affiliations:** 1 Department of Biology, Kenyon College, Gambier, Ohio, United States of America; 2 Department of Bioengineering, Tokyo Institute of Technology, Yokohama, Japan; 3 National Institute of Diabetes and Digestive and Kidney Diseases, National Institutes of Health, Bethesda, Maryland, United States of America; University of Groningen, The Netherlands

## Abstract

**Background:**

The TolC outer membrane channel is a key component of several multidrug resistance (MDR) efflux pumps driven by H^+^ transport in *Escherichia coli*. While *tolC* expression is under the regulation of the EvgA-Gad acid resistance regulon, the role of TolC in growth at low pH and extreme-acid survival is unknown.

**Methods and Principal Findings:**

TolC was required for extreme-acid survival (pH 2) of strain W3110 grown aerobically to stationary phase. A *tolC* deletion decreased extreme-acid survival (acid resistance) of aerated pH 7.0-grown cells by 10^5^-fold and of pH 5.5-grown cells by 10-fold. The requirement was specific for acid resistance since a *tolC* defect had no effect on aerobic survival in extreme base (pH 10). TolC was required for expression of glutamate decarboxylase (GadA, GadB), a key component of glutamate-dependent acid resistance (Gad). TolC was also required for maximal exponential growth of *E. coli* K-12 W3110, in LBK medium buffered at pH 4.5–6.0, but not at pH 6.5–8.5. The TolC growth requirement in moderate acid was independent of Gad. TolC-associated pump components EmrB and MdtB contributed to survival in extreme acid (pH 2), but were not required for growth at pH 5. A mutant lacking the known TolC-associated efflux pumps (*acrB*, *acrD*, *emrB*, *emrY*, *macB*, *mdtC*, *mdtF*, *acrEF*) showed no growth defect at acidic pH and a relatively small decrease in extreme-acid survival when pre-grown at pH 5.5.

**Conclusions:**

TolC and proton-driven MDR efflux pump components EmrB and MdtB contribute to *E. coli* survival in extreme acid and TolC is required for maximal growth rates below pH 6.5. The TolC enhancement of extreme-acid survival includes Gad induction, but TolC-dependent growth rates below pH 6.5 do not involve Gad. That MDR resistance can enhance growth and survival in acid is an important consideration for enteric organisms passing through the acidic stomach.

## Introduction

*Escherichia coli* expresses a large number of multi-drug resistance (MDR) efflux pumps for the expulsion of antibiotics and metabolic wastes. An important group of inner membrane efflux pumps interacts with the outer membrane channel TolC proteins to form complexes that traverse the inner membrane, periplasm, and outer membrane. These complexes efficiently pump the materials outside of the cell [1]–[5]. The other components of these TolC-dependent tripartite efflux systems consist of an inner membrane bound transporter such as the “resistance nodulation division” (RND) family transporter AcrB or the major facilitator superfamily (MFS) transporter EmrB, both driven by H^+^ influx, or the ABC-superfamily transporter MacB driven by ATP hydrolysis [6]. Stabilizing the transporter-channel interaction is a cognate periplasmic membrane fusion protein (MFP) such as AcrA, EmrA and MacA. Homologs of the *E. coli tolC* are important in virulence for pathogens such as *Salmonella typhimurium* [7], *Legionella pneumophila* [8], *Francisella tularensis* [9], and *Xylella fastidiosa* [10]. The TolC-dependent efflux system is responsible not only for expulsion of toxic compounds but also for export of intracellular metabolites, such as enterobactin, porphyrin, and excess cysteine [4], [11], [12].

Several pieces of evidence link *tolC* expression to acid pH resistance. TolC shows acid-enhanced expression in the *E. coli* proteome [13]. In *E. coli*, *tolC* is a member of the EvgA acid resistance regulon [14], [15] and, in *F. tularensis*, the *tolC* homolog is expressed in the same operon with *gad* (glutamate decarboxylase) [9], an important acid resistance factor (reviewed by [16], [17]). The Gad acid resistance system (AR2) is active in stationary-phase cells grown at pH 7 or pH 5.5, in contrast to the glucose-repressed CRP system (AR1) which requires induction in acid, pH 5.5 [16]. Furthermore, assembly of TolC into efflux complexes requires low pH [18]. The acid-dependent expression and MDR assembly have been suggested to explain the increased sensitivity of bacteria to many antibiotics above pH 7 [18].

Nevertheless, the role of MDR pumps in *E. coli* acid growth and survival has not been tested. For comparison, at high pH, overexpression of the drug resistance pump MdfA has been shown to increase survival, and actually extends the *E. coli* growth range to pH 10 [19]. Since enteric pathogens must pass through the stomach, it is important to know whether MDR pumps have a role in growth or survival in acid. Here we report the contributions of *tolC*, *emrB*, and *mdtB* to extreme-acid survival (viability of cells following exposure to pH 2), the requirement of TolC for normal exponential growth at moderately low external pH (pH 4.5–6.0), and the requirement of TolC for Gad expression and induction at low pH.

## Results

### Extreme-acid survival of *tolC*, *emrB*, and *mdtB*

**Figure 1:**
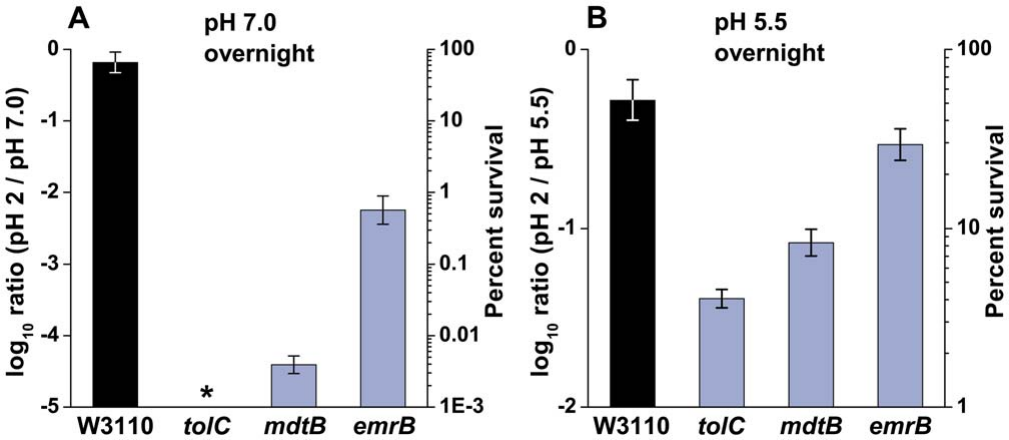
TolC, EmrB, and MdtB are required for extreme-acid survival. Strains W3110 (K-12 parent strain), JLS1015 (W3110 *tolC*::*kan*), JLS1027 (W3110 *emrB*::*kan*), and JLS1024 (*mdtB*::*kan*) were diluted into LBK pH 2 and exposed with rotation for 2 hours at 37°C after overnight growth to stationary phase in buffered LBK at **A**) pH 7.0 for non-acid-adapted cells and **B**) pH 5.5 for acid-adapted cells. Error bars = SEM, n = 6. *Below the level of detection.

TolC associates with at least nine different inner-membrane protein complexes (such as EmrAB or MdtABC) to form a connected efflux pump system [6]; several of these in the RND and MFS families, are driven by H^+^ influx. The growth and survival phenotypes of *tolC* defect strains may result directly from the absence of TolC or from the combined inactivation of several inner-membrane efflux pumps. Therefore, we investigated whether these RND and MFS transporter pump components played a role in extreme acid survival. Of the strains tested, only *tolC*, *emrB*, and *mdtB* deletions showed a significant effect on extreme-acid survival of aerobic cultures (Fig. 1). MDR deletion strains *acrB*, *emrY*, and *mdtF* showed survival levels comparable to the wild-type (data not shown). Survival was tested first for overnight cultures grown at external pH 7, where the Gad system is available but not the acid-inducible CRP system [16]. Extreme-acid survival (exposure at pH 2 for 2 hrs) was over 10^5^-fold lower for *tolC*, 10^4^-fold lower for *mdtB*, and 100-fold lower for *emrB* compared to wild-type strain W3110 (Fig. 1A). There was no increase or decrease in survival for a *marR* defective strain in which TolC expression is upregulated (data not shown) [20].

Acid survival was also tested for bacteria cultured overnight at pH 5.5, where RpoS- and CRP-dependent acid resistance systems are expressed [16]. Cultures grown at external pH 5.5 showed a 13-fold decrease in survival of *tolC* compared to W3110 (Fig. 1B). Thus, the TolC requirement was much greater for cells grown at pH 7 than for cells grown at pH 5.5. Complementation of *tolC* with plasmid pMX, which produces a functional TolC, grown at pH 5.5 and challenged at pH 2 restored the strain's acid survival comparable to that of the wild-type (data not shown). Strains defective for *mdtB* and *emrB* showed only a 6-fold and 2-fold decrease in survival under these conditions, respectively.

In extreme base (pH 10), the *tolC* strain (cultured aerobically to stationary phase at pH 8) showed comparable survival to the wild-type (data not shown). Thus, the pH sensitivity of *tolC* mutants was limited to acidic pH.

### TolC is required for expression of the glutamate-dependent acid resistance system

A major contribution to acid resistance can result from the glutamate decarboxylase (Gad) system encoded by *gadA* and *gadBC*. The *gadA* and *gadB* genes encode isoforms of glutamate decarboxylase and *gadC* encodes the glutamate/γ-aminobutyric acid antiporter [16]. Survival of strains MG1655 (wild-type) and MG1655T (*tolC*::Tn*10*) grown in LBK at pH 5.5 (100 mM MES) overnight and exposed to low external pH (pH 2.5) was tested in M9-glucose medium supplemented with 1.5 mM L-glutamic acid. After 30 min, survival of the *tolC* strain was decreased 10-fold relative to the wild type; and after 60 min, survival of the *tolC* strain dropped to nearly 20-fold below the wild-type (data not shown). This is comparable to the acid-survival seen in complex LB medium (Fig. 1B) and suggests that the *tolC* strain is unable to utilize the glutamate-dependent acid resistance system, which is expressed during the overnight growth before exposure to pH 2.

**Figure 2:**
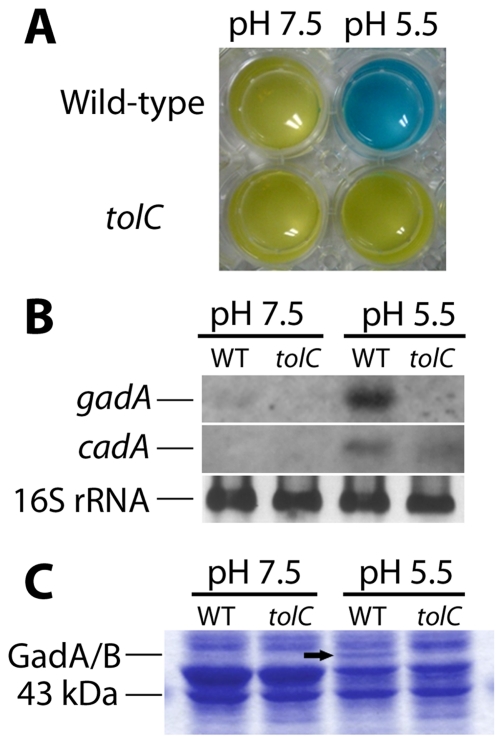
TolC is required for expression of the glutamate-dependent acid resistance system. *E. coli* wild-type MG1655 and its TolC-deficient derivative MG1655T (*tolC*::Tn*10*) were assayed for glutamate decarboxylase activity, *gadA* mRNA expression, and GadA/B expression. **A**) Both strains were tested for glutamate decarboxylase activity at pH 7.5 and pH 5.5 using the GAD reagent as described in Materials and Methods. The pH indicator dye bromocresol green changes from yellow to blue when L-glutamic acid is decarboxylated. There was almost no change in color with the *tolC*::Tn*10* strain at pH 5.5. **B**) Northern analysis of *gadA* and *cadA* mRNA at pH 7.5 and pH 5.5 with the wild-type and *tolC*::Tn*10* strains; 16S rRNA bands are provided as a control. **C**) A 53 kDa protein band (indicated by an arrow) was observed by SDS-PAGE only in wild-type cultures at pH 5.5; this band was identified as a mixture of GadA and GadB proteins by mass spectrometry analysis (MALDI-TOF).

Cultures of MG1655 and MG1655T (*tolC*::Tn*10*) were also assayed for activity and expression of the Gad system. Glutamate decarboxylase activity was assessed using the pH indicator dye bromocresol green; the dye changes from yellow to blue upon pH increase in the reaction mixture, following decarboxylation of L-glutamate (Fig. 2A). The wild-type strain behaved as expected with no decarboxylation at pH 7.5 and very clear evidence of glutamate decarboxylation at pH 5.5. The *tolC* strain, however, showed almost no Gad activity at pH 5.5. The *gadA* mRNA transcription was observed in wild-type but not in *tolC* cultures at pH 5.5, whereas the mRNA transcript of the lysine-dependent acid resistance system (*cadA*) was present in both wild-type and *tolC* strains (Fig. 2B). In the *tolC* strain at pH 5.5, the *cadA* mRNA transcript showed decreased expression compared to the wild-type, which may result from decreased regulation by GadE [21]. Both GadA and GadB proteins were absent in the *tolC* strain at pH 5.5 (Fig. 2C). Without TolC, no *gadA* or *gadB* expression could be detected.

Gad expression was restored by the pMX plasmid carrying the wild-type *tolC* gene (data not shown). Thus, TolC is required for expression of *gadA* mRNA and GadA and GadB proteins, as well as for activity of glutamate decarboxylases. Furthermore, in a *tolC* defective strain, plasmids expressing either GadB-C (pMF565) or the positive regulator GadE (pQEgadE) each restored extreme-acid survival at pH 2. This complementation confirms the role for Gad in the TolC requirement for extreme-acid survival.

### Extreme-acid survival in a multiple-MDR efflux pump mutant

**Figure 3:**
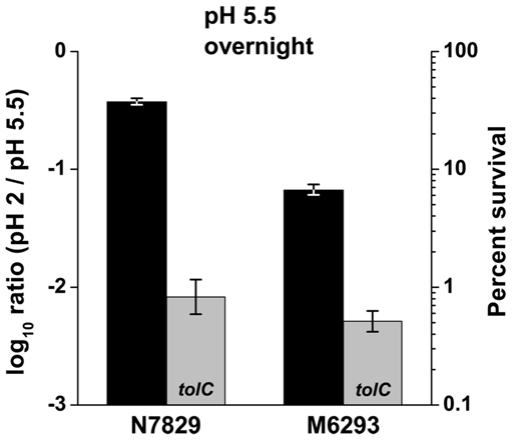
TolC is required for extreme-acid survival in a strain defective for eight MDR efflux inner membrane pumps. Strains N7829 (K-12 derivative), M5567 (N7829 *tolC*::*kan*), M6293 (N7829 *acrB*::*frt acrD*::*frt emrB*::*frt emrY*::*frt macB*::*frt mdtC*::*frt mdtF*::*frt acrEF*::*spc*), and JLS1042 (M6293 *tolC*::*kan*) were grown overnight to stationary phase in LBK buffered at pH 5.5 (100 mM MES), diluted into LBK pH 2, and exposed for 2 hours at 37°C. Grey bars represent the parent strain listed with the additional kanamycin resistance insertion in *tolC*. Error bars = SEM, n = 6.

TolC acts as the outer-membrane conduit for export by several inner-membrane efflux pump complexes [5]. We investigated whether loss of several TolC-dependent pump complexes would affect acid resistance in a manner comparable to loss of TolC. Extreme-acid survival was tested for strain M6293, which is defective for the inner-membrane pumps of eight known TolC-dependent MDR efflux complexes (AcrAB, AcrAD, AcrEF, EmrAB, EmrKY, MacAB, MdtABC, MdtEF). Acid survival of the multi-pump defective strain was compared to its parent strain N7829, and to strains deleted for *tolC* grown to stationary phase at pH 5.5 (Fig. 3). The parent strain N7829 survived the acid challenge as well as other wild-type *E. coli* K-12 strains. M6293, the strain lacking the TolC-associated efflux pumps, including EmrB and MdtC, showed a 6-fold decrease in survival versus the parent strain N7829. This result is comparable to the survival percentages seen with strains lacking MdtB (6-fold) or EmrB (2-fold) in Fig. 1B. When *tolC* was also disrupted in these two strains, survival was decreased to below 1%. A strain with defects in both the EmrAB and MdtABC complexes (W3110 *emrB*::*frt mdtB*::*kan*) showed 4- to 10-fold decrease in survival (data not shown).

### pH-dependent growth of *tolC* defective strains

**Figure 4:**
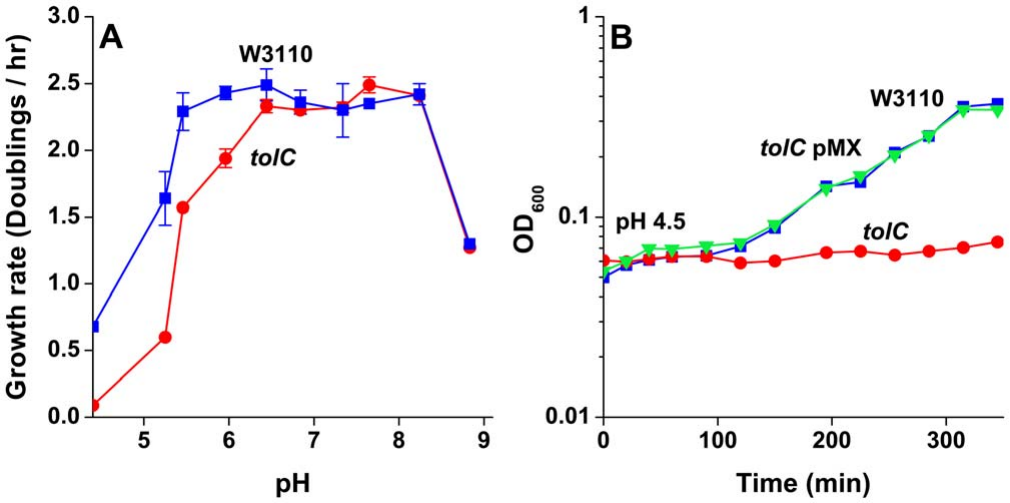
TolC is required for acid growth. **A**) Strains were grown from pH 4.5–pH 9.0 using the appropriate buffer in half unit increments at 37°C. W3110 (blue) and *tolC*::*kan* (red) growth rates calculated in early log-phase (OD_600_ 0.1 to 0.3) are depicted as a function of pH. **B**) Successful complementation of the *tolC* strain with pMX, a low-copy plasmid that carries the functional *tolC* gene (green), in LBK at pH 4.5 (100 mM HOMOPIPES) restored the acid growth capability to that of the wild type W3110 (blue). Cultures were maintained as described in the Materials and Methods. Error bars = SEM (n = 3) and absent when smaller than the symbol.

While numerous genes are known to affect survival at pH 2, relatively few affect exponential growth at moderately low pH. The best studied case is the triple potassium transport deletion which results in K^+^-dependent growth at low pH [22]. To determine the role of TolC in acidic growth, we assessed the ability of a strain defective for *tolC* to grow in LBK with an external pH range of 4.5–9.0 (Fig. 4A). At pH 4.5, the growth rate of *tolC* was near zero. Over the range of pH 4.5–6.0, the *tolC* strain grew at a slower rate than the parent. Over the range of external pH 6.5–9.0, there was no significant difference in growth; thus, the effect of the deletion of *tolC* on growth rate was limited to the range of acid stress. Wild-type growth at pH 4.5 was restored by complementation of the *tolC* strain with the *tolC*-carrying plasmid pMX (Fig. 4B). The *tolC* defect had no effect on cytoplasmic pH when cultures were suspended at pH 4.5 to pH 6.0 (data not shown), using GFPmut3b fluorimetry as described previously [22], [23].

The pH-dependent growth of the *tolC* strain could be caused by a defect in the channel connection to one or more of its associated inner-membrane efflux complexes [24]. Growth at pH 5 was tested for a mutant deleted for the known TolC-dependent efflux porters (N7829 *acrB*::*frt acrD*::*frt emrB*::*frt emrY*::*frt macB*::*frt mdtC*::*frt mdtF*::*frt acrEF*::*spc*). No difference was seen between the growth of mutant and parent (data not shown).

Given that a *tolC* strain does not appear to express the glutamate-dependent acid resistance system, we assessed the contribution of the Gad system to the pH-sensitive growth of a *tolC* mutant. The antiporter GadC imports glutamate in exchange for the decarboxylation product [16]. When a *gadC tolC* double mutant (JLS1048) was grown in early log-phase at pH 5, growth rates similar to the *tolC* strain were observed, while the wild-type W3110 and a strain lacking *gadC* had comparable growth rates that were higher than the *tolC* strains (data not shown). No growth defect was seen in a *gadC* strain at pH 5.0; this finding is consistent with previous studies showing that the Gad regulon is needed only for extreme acid survival, not for growth in moderate acid [16]. Furthermore, growth rates in *tolC* cultures grown at pH 5 were unaffected by expression of GadE or GadB-C produced by plasmids pQEgadE and pMF565, respectively (data not shown), indicating that even over-expression of Gad system components on a low-copy plasmid could not restore wild-type growth rates. Thus, the poor growth at moderately low pH in *tolC* strains must involve a mechanism other than TolC-mediated induction of the glutamate-dependent acid resistance system.

## Discussion

TolC is a component of several MDR efflux complexes that enable *E. coli* to expel both toxins and metabolic wastes across the periplasm and outer membrane, driven by H^+^ antiport [5]. Expression of TolC is regulated by MarA, SoxS and Rob [20] and by the EvgA acid resistance regulon [14], which suggests that TolC may function in acid adaptation. Consistent with acid adaptation, TolC-associated drug efflux of toxins is more active at low pH [25]. Our finding that TolC contributes to acid resistance is the first report of an MDR pump component that enhances acid adaptation. For comparison, at high pH over-expression of MDR pump MdfA confers alkali-tolerance and extends the upper pH range for growth [19]. Our results suggest a general possibility that when antibiotics select for gain of MDR pumps by enteric bacteria, the bacteria may show increased resistance to stomach acid.

Additionally, two TolC-associated inner-membrane MDR efflux pump components, EmrB and MdtB, contributed to extreme-acid survival. MdtABC comprises RND-family efflux pumps and a membrane fusion protein which, along with *tolC*, is under the regulation of the BaeR regulon [26]–[28]. The BaeSR two-component regulatory system is an envelope stress signaling pathway that responds to extracytoplasmic stress, which may include acidic pH [27], [29]. EmrB is a major facilitator superfamily MDR efflux pump that is induced by permeant weak acids, such as salicylate [30]. Other TolC-associated inner membrane pumps that were tested for extreme-acid survival, such as AcrB, EmrY, and MdtF, showed comparable survival to that of the wild-type.

Of all MDR efflux pump components tested, TolC contributes the most to extreme-acid survival at pH 2, even in a background strain that lacks eight TolC-dependent inner membrane pumps (Fig. 3). While the multiple MDR efflux pump mutant pre-grown at pH 5.5 showed 6-fold decreased survival at pH 2 compared to its wild-type, a *tolC* deletion in either the parent strain or the MDR mutant showed a much larger decrease (45- and 12-fold, respectively; Fig. 3). Thus, either TolC itself plays a major role in extreme-acid survival independent of its associated inner-membrane pumps, or else an unidentified TolC-dependent pump is involved.

The mechanism of the TolC effect in acid survival was shown to include regulation of the Gad system. The decarboxylation of glutamate by GadA and GadB is one of the main pH homeostasis mechanisms active at low external pH and in stationary phase; cells lacking this system are unable to maintain cytoplasmic pH and perform poorly when challenged in acidic media [16]. In a strain lacking TolC grown at pH 5.5, we identified almost no glutamate decarboxylase activity (Fig. 2A), observed no *gadA* mRNA among total RNA isolated (Fig. 2B), and detected no GadA and GadB protein expression (Fig. 2C). Thus, TolC is required for induction of the glutamate-dependent acid-resistance system. The restoration of pH 2 survival by GadB-C or by GadE provided on a plasmid confirms that the TolC requirement involved Gad regulation. This may explain why the TolC requirement for acid survival was greatest for cells grown at pH 7 (Fig. 1A) where the acid-induced AR1 system thatinvolves CRP is unavailable, and thus Gad offers the main mechanism of acid resistance [16].

On the other hand, the requirement for TolC for exponential growth in moderate acid (pH 4.5–6.0) was shown to be independent of Gad. Deletion of *gadC* significantly reduces extreme-acid survival (pH 2.5) [31], [32], verifying that a *gadC* deletion inactivates Gad activity. Nevertheless, a *gadC* deletion strain did not exhibit a growth defect during exponential growth in moderate acid and expression of the positive regulator GadE or GadB-C from plasmids did not restore wild-type growth rates in a *tolC* mutant strain (LBK, pH 5.0; data not shown). Thus, while the role of TolC in Gad regulation may be the reason TolC is required for extreme-acid survival, the decreased growth rates of the *tolC* strain only in acidic conditions are not the result of a lack of Gad activity. TolC was needed for maximal growth below pH 6.5, where cytoplasmic pH is less than optimal (pH 7.4–7.8) (Fig. 4).

The growth defect and the acid resistance defect of the *tolC* strain were both complemented with a complete *tolC* gene on a low copy plasmid. The complementing plasmid pMX does not carry the *ygiABC* genes downstream from *tolC* that may be in the *tolC* operon [33]. Thus, the low pH growth effect is not due to YgiABC activity. Complementation confirms that *tolC*, and not adjacent genes, contributes to acid resistance.

Our findings suggest a novel physiological role for TolC in pH homeostasis in acidic conditions. Previous reports demonstrate no growth defects in LB medium, but find impaired cell division and growth in minimal glucose medium [33]. The requirement of TolC for growth at low pH is surprising because TolC resides in the outer membrane, mediating exchange of the external medium with the periplasm; and the periplasmic pH generally equals the external pH [23]. Thus it is hard to see why cytoplasmic pH homeostasis would require an outer-membrane channel. A possibility is that products excreted during metabolism at low external pH accumulate in the periplasm, if they cannot be removed without the TolC channel.

The mechanism of TolC may or may not involve its interactions with the inner membrane efflux pumps [24]. The fact that deletion of eight major inner-membrane efflux pumps has no effect on growth and a relatively modest effect on extreme-acid survival of pH 5.5-grown cultures (Fig. 3) suggests that the significant reduction of extreme-acid survival in *tolC* deletion strains is independent of the channel's association with these pumps. The proton motive force from the periplasm to the cytoplasm drives the functioning of many multidrug efflux transporters [34]. In addition to functioning as an outer membrane pore for many MDR pumps, TolC may also play a physiological role in pH homeostasis through an interaction with the proton motive force that drives efflux. The original function of TolC may have been to provide the cell with a pH homeostasis mechanism in acidic conditions that later was co-opted to function as a common outer membrane porin in multidrug resistance.

As we completed our manuscript, we became aware of an unpublished plate screen showing that *E. coli* colony growth at low pH requires several envelope and inner membrane components besides TolC, such as TolB and TolR; the report has since been published [35]. We have since confirmed with quantitative growth curves and survival assays the low-pH specific growth requirements for TolB and TolR (G. Garduque and J. Slonczewski, unpublished). It will be of interest to determine how all these envelope components relate to pH homeostasis.

## Materials and Methods

### Bacterial strains, media, and growth conditions

The *E. coli* K-12 strains used here are described in Table 1. W3110 [36] was used as the wild-type strain unless indicated otherwise. Deletion strain M6293 (N7829 *acrB*::*frt acrD*::*frt emrB*::*frt emrY*::*frt macB*::*frt mdtC*::*frt mdtF*::*frt acrEF*::*spc*) was compared to parental strain N7829 (GC4468). Deletion alleles containing a kanamycin resistance insertion (Km^R^) were transduced from the Keio collection [37], obtained from the Coli Genetic Stock Center (Yale University), into the wild-type strain by P1 phage transduction. “*frt*” is the designation for the “scar” sequence remaining at the site of the cured Keio *kan* insertion. Deletion strains were maintained with 50 µg/ml kanamycin. Plasmid pMX carrying the wild-type *tolC* gene on a low-copy-number vector pMW119 (derived from pSC101) was transformed into JLS1015 (W3110 *tolC*::*kan*) for complementation experiments [4]. Strains containing plasmid pMX were maintained with 50 µg/ml ampicillin in overnight cultures and 20 µg/ml ampicillin in growth cultures.

**Table 1:** *E. coli* K-12 strains and plasmids used in this study.

Strain or plasmid	Genotype	Source
W3110	K-12 (F^−^ λ^−^)	[36]
JLS9318	MC4100 *gadC*::Tn*10*	[31]
JLS1015	W3110 *tolC732*::*kan* (Keio JW5503)	This work
JLS1023	W3110 *acrB747*::*kan* (Keio JW0451)	This work
JLS1024	W3110 *mdtB774*::*kan* (Keio JW2060)	This work
JLS1025	W3110 *mdtF769*::*kan* (Keio JW3482)	This work
JLS1026	W3110 *emrY776*::*kan* (Keio JW2364)	This work
JLS1027	W3110 *emrB767*::*kan* (Keio JW2661)	This work
JLS1036	W3110 *tolC732*::*kan* pMX	This work
JLS1039	W3110 *marR751*::*kan* (Keio JW5248)	This work
JLS1048	W3110 *tolC732*::*kan gadC*::Tn*10*	This work
JLS1050	W3110 *emrB767*::*frt mdtB774*::*kan*	This work
MG1655	K-12	M. Wachi
MG1655T	MG1655 *tolC*::Tn*10*	This work
N7829	K-12; GC4458	J.L. Rosner
M5567	N7829 *tolC732*::*kan*	J.L. Rosner
M6293	N7829 *acrB747*::*frt acrD790*::*frt emrB767*::*frt emrY776*::*frt macB780*::*frt mdtC775*::*frt mdtF769*::*frt acrEF*::*spc*	J.L. Rosner
JLS1042	M6293 *tolC732*::*kan*	This work
Plasmids		
pMX	pMW119 carrying the *tolC* gene	[41]
pQEgadE	pQE80L carrying the *gadE* gene	[44]
pMF565	pQE80L [*gadBC*]his-*gadB* Ap^R^	J.W. Foster

Bacteria were cultured in LBK medium (10 g/l tryptone, 5 g/l yeast extract, and 7.45 g/l KCl) supplemented with pH buffers as needed [38]. Overnight cultures of deletion strains were maintained with kanamycin (50 µg/ml). Media were buffered with 100 mM Homopiperazine-N, N′-bis-2-(ethanesulfonic acid) (HOMOPIPES; pKa = 4.55), 2-(N-morpholino) ethanesulfonic acid (MES; pKa = 5.96), 1,4-Piperazinebis(ethanesulfonic acid) (PIPES; pKa = 6.66), 3-(N-morpholino)propanesulfonic acid (MOPS; pKa = 7.01), N-Tris(hydroxymethyl)methyl-3-aminopropanesulfonic acid (TAPS; pKa = 8.11), 3-[(1,1-Dimethyl-2-hydroxyethyl)amino]-2-hydroxypropanesulfonic acid (AMPSO; pKa = 9.10), or 3-(Cyclohexylamino)-1-propanesulfonic acid (CAPS; pKa = 10.08). At the end of the experiments, the pH of the cultures was checked to ensure that it was within 0.2 pH units of the original uninoculated medium.

### Acid and base resistance assays

The conditions for testing acid resistance (survival in extreme acid) of aerated cultures were based on those previously described, with modifications [39], [40]. Cells were cultured with rotary aeration overnight (16–18 hr at 37°C) to stationary phase in LBK pH 5.5 (100 mM MES) or LBK pH 7 (100 mM MOPS). Overnight cultures were diluted 200-fold into LBK pH 2 and incubated with rotation at 37°C. Following a 2 hr exposure, cultures were serially diluted and plated on LBK-agar. Overnight cultures were also diluted 200-fold into LBK 100 mM MOPS, pH 7 and immediately serially diluted and plated onto LBK-agar. Plates were incubated overnight at 30°C.

Percent survival was calculated as follows: since acid survival represents an exponential death curve, colony counts of surviving cells and control plates were log_10_-transformed to provide a normal distribution of the data. The mean of the unexposed controls was then subtracted from the mean of exposed pH 2 colony counts, resulting in a log_10_ ratio that correlates to percent survival. All errors stated are the standard error of the mean (SEM). Each experimental condition consisted of six biological replicates from the same overnight culture. Each entire experiment was conducted at least twice.

For base resistance (survival in extreme base), bacteria were cultured with aeration in LBK pH 8.0 (100 mM TAPS) and diluted into LBK pH 10 (100 mM CAPS). Survival was measured and calculated as for acid resistance.

### Glutamic acid decarboxylase assays

*E. coli* K-12 derivative strains MG1655 and MG1655T (*tolC*::Tn*10*), transduced by P1-phage from JA300T [41], were used in the assessment of glutamic acid decarboxylase activity. Glutamic acid decarboxylase activity was assessed using the GAD reagent (1 g/l L-glutamic acid, 0.05 g/l bromocresol green, 90 g/l NaCl, 3 ml/l Triton X-100) with minor modifications [42]. MG1655 and MG1655T cultures were grown for 1 hr in LB (pH 7.5±0.2) or LB buffered with 100 mM MES (pH 5.5). Cells were harvested, washed with saline (0.85% NaCl), and suspended in the same solution. An aliquot of cell suspension (9×10^8^ cells) was transferred to a new tube, and 1 ml of the GAD reagent was added. The reaction mixtures were incubated for 1 hr at 35°C and then evaluated for decarboxylase activity by a color change from yellow to blue.

The presence of *gadA* and *cadA* mRNA in both MG1655 and MG1655T at pH 7.5 and pH 5.5 was assessed using Northern analysis. Total cellular RNA was isolated using the RNeasy kit (Qiagen) and separated by formaldehyde-agarose gel electrophoresis. Hybridization was done with the DIG luminescent detection kit (Roche Dignostics).

To assess the presence of GadA and GadB proteins, cultures of MG1655 and MG1655T were grown in LB medium at pH 5.5 (100 mM MES) and pH 7.5. Cells were harvested, suspended in a 50 mM sodium phosphate buffer (pH 7.0), and disrupted by sonication. After unbroken cells were removed, lysate proteins were separated by SDS-polyacrylamide gel electrophoresis (10% acrylamide) and stained with Coomassie Brilliant Blue. The 53-kDa protein band was cut out and analyzed by mass spectrometry (MALDI-TOF/TOF ultrafleXtreme, Bruker Daltonics).

Glutamate-dependent extreme-acid resistance was tested with overnight cultures grown in LB buffered with 100 mM MES pH 5.5, then diluted into warmed M9 medium (6.8 g/l Na_2_HPO_4_, 3.0 g/l KH_2_PO_4_, 0.5 g/l NaCl, 1.0 g/l NH_4_Cl, 2 mM MgSO_4_, 0.1 mM CaCl_2_, and 0.4% glucose) supplemented with 1.5 mM L-glutamic acid and adjusted to pH 2.5. Surviving cells were counted after 30 and 60 min of acid challenge as previously described [43].

### Acid growth assays

To test acid growth, cells were cultured with aeration to stationary phase (16–18 hr, 37°C) in unbuffered LBK. Overnight cultures were diluted 100-fold into LBK pH 4.5–9.0 (in half unit increments) including 100 mM of the pH-appropriate buffer, and rotated at 37°C until cultures reached stationary phase. OD_600_ was measured at regular intervals after the initial dilution. Growth rates were calculated as doublings per hour over the region of exponential growth (approximately OD_600_ = 0.1 to 0.3). The wild-type strain W3110 and its *tolC* derivative (JLS1015) were also tested for loss of cytoplasmic pH homeostasis at low pH as described previously [22], [23].
